# Sequencing the *CaSR* locus in Pakistani stone formers reveals a novel loss-of-function variant atypically associated with nephrolithiasis

**DOI:** 10.1186/s12920-021-01116-5

**Published:** 2021-11-12

**Authors:** Ihsan Ullah, Isabel Ottlewski, Wasim Shehzad, Amjad Riaz, Sadaqat Ijaz, Asad Tufail, Hafiza Ammara, Shrikant Mane, Shirlee Shril, Friedhelm Hildebrandt, Muhammad Yasir Zahoor, Amar J. Majmundar

**Affiliations:** 1 Molecular Biology Section, Institute of Biochemistry and Biotechnology, University of Veterinary and Animal Sciences, 54000 Lahore, Pakistan; 2 Department of Pediatrics, Boston Children’s Hospital, 300 Longwood Avenue, MA 02115 Boston, USA; 3 Department of Theriogenology, University of Veterinary and Animal Sciences, Lahore, Pakistan; 4 Department of Genetics, Yale University School of Medicine, New Haven, CT USA; 5 Yale Center for Mendelian Genomics, Yale University School of Medicine, New Haven, CT USA

**Keywords:** Calcium sensing receptor, *CaSR*, Nephrolithiasis, Rare disease

## Abstract

**Background:**

Nephrolithiasis (NL) affects 1 in 11 individuals worldwide and causes significant morbidity and cost. Common variants in the calcium sensing receptor gene (*CaSR*) have been associated with NL. Rare inactivating *CaSR* variants classically cause hyperparathyroidism, hypercalcemia and hypocalciuria. However, NL and familial hypercalciuria have been paradoxically associated with select inactivating *CaSR* variants in three kindreds from Europe and Australia.

**Methods:**

To discover novel NL-associated *CaSR* variants from a geographically distinct cohort, 57 Pakistani families presenting with pediatric onset NL were recruited. The *CaSR* locus was analyzed by directed or exome sequencing.

**Results:**

We detected a heterozygous and likely pathogenic splice variant (GRCh37 Chr3:122000958A>G; GRCh38 Chr3:12228211A>G; NM_000388:c.1609-2A>G) in *CaSR* in one family with recurrent calcium oxalate stones. This variant would be predicted to cause exon skipping and premature termination (p.Val537Metfs*49). Moreover, a splice variant of unknown significance in an alternative *CaSR* transcript (GRCh37 Chr3:122000929G>C; GRCh38 Chr3:122282082G >C NM_000388:c.1609-31G >C NM_001178065:c.1609-1G >C) was identified in two additional families.

**Conclusions:**

Sequencing of the *CaSR* locus in Pakistani stone formers reveals a novel loss-of-function variant, expanding the connection between the *CaSR* locus and nephrolithiasis.

**Supplementary Information:**

The online version contains supplementary material available at 10.1186/s12920-021-01116-5.

## Introduction

Nephrolithiasis (NL) affects 1 in 11 individuals during their lifetime [1, 2]. NL is associated with significant patient morbidity, recurrence, and healthcare costs [3]. The causes of NL are not well understood. Formerly, monogenic causes of NL were thought to be restricted to rare tubulopathies and genetic syndromes. However, we demonstrated that a causative monogenic mutation can be detected in one of 30 known NL causing genes in ~ 6 to 11% of adult and ~ 17 to 29% pediatric cases [4–7].

The calcium sensing receptor (*CaSR*) gene regulates calcium homeostasis [8], and rare variants in *CaSR* cause calcium disorders [9, 10]. Inactivating *CaSR* variants are associated with hyperparathyroidism, hypercalcemia, and hypocalciuria with dominant (OMIM: 145980) and recessive (OMIM: 239200) modes of inheritance [10]. Heterozygous activating *CaSR* variants cause autosomal dominant hypoparathyroidism, hypocalcemia and hypercalciuria (OMIM: 601198) [9, 11].

NL has been associated with common *CaSR* variants, which cause either increased activity or decreased expression [12–27]. NL is less typically the presenting problem in patients with calcium disorders caused by rare *CaSR* variants. 3.5% of cases with activating *CaSR* variants have NL or nephrocalcinosis, but this is typically an iatrogenic complication of treatment [28]. NL has also been observed in three atypical kindreds from Europe and Australia with dominant inactivating variants (L650P, F881L, K336del), hypercalcemia, and familial hypercalciuria [29, 30]. These kindreds suggest that the functions of the calcium sensing receptor in serum calcium homeostasis and parathyroid hormone regulation can be uncoupled from its role in renal calcium excretion. Nevertheless, these position-specific inactivating variants are exceedingly rare, given that a deleterious *CaSR* variant was not detected in a worldwide cohort of 697 NL families using gene panel or exome sequencing [4–7]. Therefore, discovery of novel NL-associated alleles can provide a deeper understanding of the role of *CaSR* in calcium handling and kidney stone disease.

To uncover novel NL-associated variants, 57 Pakistani families presenting with pediatric onset NL were recruited. The *CaSR* locus was analyzed by directed sequencing in 20 families (47 individuals) and from exome sequencing data in an additional 37 families (46 individuals). We detected a novel heterozygous and likely pathogenic splice variant (NM_000388:c.1609-2A>G) in *CaSR* in one family with recurrent calcium oxalate stones. This variant would be predicted to cause skipping of exon 6 and premature termination (p.Pro537Metfs*49). Moreover, a splice variant of unknown significance in an alternative *CaSR* transcript (NM_001178065:c.1609-1G>C) was identified in two additional families. In summary, sequencing of the *CaSR* locus in Pakistani stone formers reveals a novel loss-of-function variant atypically associated with nephrolithiasis.

## Methods

### Subject recruitment and DNA sample extraction

The period of recruitment was 01/2013 to 08/2017. Subjects were evaluated by a Urologist for an NL episode at the Lahore General Hospital, Services Hospital, and associated clinics in Punjab, Pakistan. For inclusion, subjects presented with evidence of NL on ultrasonography and had onset of NL before age 21 years. Results of stone analyses (performed by Fourier transform infrared spectroscopy) were recovered where available. Subjects were excluded if the primary physician determined that the NL episode was potentially caused by an underlying medical condition (gastrointestinal disease, diuretic use), primary non-calcium metabolic disorder (e.g. primary or secondary hyperoxaluria), or cystic kidney disease (e.g. the presence of at least one renal cyst). At enrollment, informed consent, clinical data, pedigree information, and DNA samples from subjects were collected.

Blood samples from affected families were stored in ethylenediaminetetraacetic acid (EDTA) vacutainers, and DNA was extracted from these samples using an organic extraction method. DNA was stored in double-distilled deionized water or tris(hydroxymethyl)aminomethane (Tris) EDTA (TE) buffer (10 mM Tris and 0.1 mM EDTA).

### Directed *CaSR* gene amplification

All coding exons and flanking regions of the *CaSR* gene were amplified by PCR using 15 pairs of primers. The annealing temperature, amplification product size, and PCR optimization conditions for each primer are given in Additional file 5: Table S1 and previously described [31]. These amplified products were sequenced using the Sanger sequencing method. The sequencing data was analyzed using the CLC Genomics Workbench (version 6.5.2) software (CLC Bio, Aarhus, Denmark). The data was aligned to the *CaSR* reference genome sequence (chr3:121,902,530–122,005,342 forward strand; GRCh37:CM000665.1). Variants from the reference genome were analyzed as shown in Additional file 1: Figure S1A and as described in the Variant Analysis section below.

### Exome sequencing

Genomic DNA was isolated from blood lymphocytes and subjected to exome capture using Agilent SureSelect (Santa Clara, CA) and human exome capture arrays (Life Technologies, Carlsbad, CA) followed by next-generation sequencing on the Illumina HiSeq sequencing platform (San Diego, CA). Sequence reads were mapped to the human reference genome assembly (NCBI build 3/hg19) using CLC Genomics Workbench version 6.5.2 software (CLC bio, Aarhus, Denmark). Following alignment to the human reference genome, variants within *CaSR* were filtered as previously described [6], and as described in the Variant Analysis section below, and in Additional file 1: Figure S1B. In any families with rare potentially deleterious variants, the exome data was further analyzed for causative variants in thirty established known NL-disease genes as previously described [6].

### Variant analysis

Variants were filtered as followed (see Additional file 1: Figure S1). First, different population databases were queried to include only rare alleles (minor allele frequency < 1%) including the Exome Sequencing Project (http://evs.gs.washington.edu/EVS), Exome Aggregation Consortium (http://exac.broadinstitute.org), Genome Aggregation Database (gnomAD; http://gnomad.broadinstitute.org), and 1000 Genomes Project (http://www.internationalgenome.org/1000-genomes-browsers). Synonymous and intronic variants that were not located within the splice site regions were excluded. Surviving variants were then ranked based on their likelihood to cause disease, taking into consideration evolutionary conservation among orthologs using the ENSEMBL Genome Browser (http://www.ensembl.org), and assembled using Clustal Omega (https://www.ebi.ac.uk/Tools/msa/clus-talo/). Three web-based programs were used to establish pathogenicity prediction scores of missense variants: Mutation Taster (http://www.mutationtaster.org), SIFT (http://sift.bii.a-star.edu.sg/), and PolyPhen-2 (http://genetics.bwh.harvard.edu/pph2). Splice variants were evaluated using the web-based programs MaxEnt (http://hollywood.mit.edu/burgelab/maxent/Xmaxentscan_scoreseq.html), NNSPLICE (https://www.fruitfly.org/seq_tools/splice.html), and splice site finder (http://www.umd.be/searchSpliceSite.html). The remaining variants were further evaluated by reviewing the existing literature and determining the phenotypic match. We consulted clinician-scientists and geneticists who are knowledgeable about the clinical phenotypes and pedigree structure and are experienced in identifying exome level mutations. In addition, the ACMG guidelines for variant classification were applied, and variants were disease-causing if they were classified as pathogenic or likely pathogenic.

## Results

### Pediatric nephrolithiasis cohort characteristics

We recruited 57 families with pediatric nephrolithiasis. 31 probands were female, while 26 were male. 23 probands (40.4%) were born from a consanguineous union based on self-reporting that parents were first-degree cousins.

### Directed and exome sequencing of *CaSR* locus

**Fig. 1:**
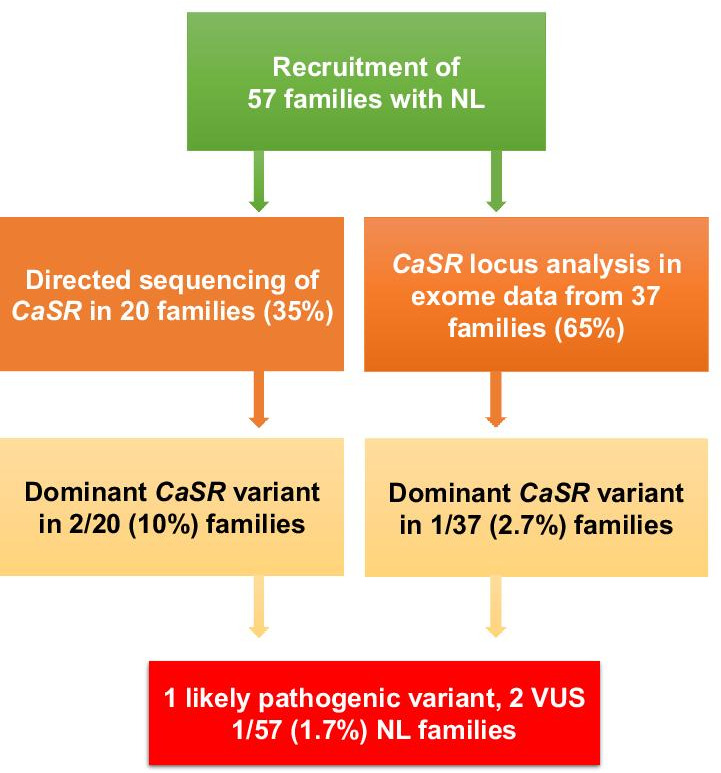
Sequencing of *CASR* locus in Pakistani families with nephrolithiasis identifies a likely pathogenic variant. Flow diagram of *CaSR* locus sequencing through directed Sanger sequencing and exome sequencing in a cohort of 57 Pakistani families with nephrolithiasis is shown. We identified dominant rare *CaSR* variants 3 of 57 (5.3%) of families, of which one is likely pathogenic and two are variants of unknown significance (VUS)

We performed directed sequencing of *CaSR* coding regions and adjacent splice sites in the first 20 families (47 individuals) with pediatric NL (Fig. 1). We identified rare variants within coding or splice regions in two families (KS-72 and KS-6). We, subsequently, queried exome sequencing data from an additional 37 Pakistani families (46 families) with pediatric NL and detected a rare variant within a splice region within one family (KS-71) (Fig. 1).

**Fig. 2:**
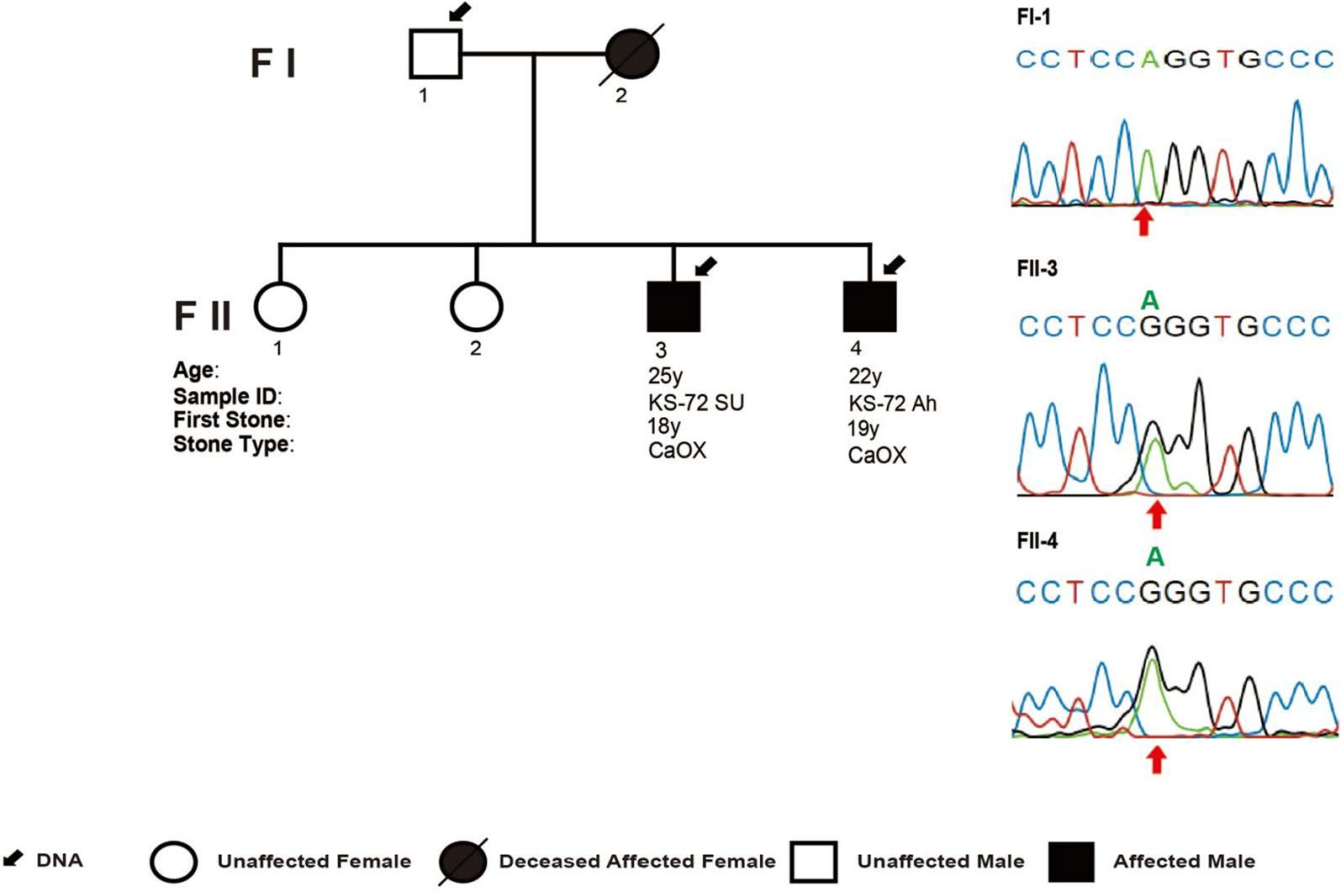
Pedigree structure of Pakistani NL family with likely pathogenic *CaSR* variant. The pedigree for family KS-72 is shown. Segregation of the likely pathogenic *CaSR* variant (NM_000388:c.1609-2A>G) is displayed on right in two affected siblings and lack of segregation with an unaffected parent by Sanger sequencing chromatograms. Clinical data is shown below each symbol, and legend is provided at bottom for pedigree

In family KS-72, there were three affected family members (Fig. 2; Table 1). The deceased mother in this pedigree had a history of NL without further clinical or genetic data. Her two sons developed bilateral NL with onset at age 18 years (II-3) and 19 years (II-4). Both had two stone episodes thus far in their lifetime. Stone composition analysis for subjects II-3 and II-4 revealed predominantly calcium oxalate nephrolithiasis (Table 1). Targeted sequencing detected a deleterious essential splice site variant (GRCh37 Chr3:122000958A>G; GRCh38 Chr3:12228211A>G; NM_000388:c.1609-2A>G; NM_001178065:c.1637A>G) in *CaSR* (Fig. 2, Additional file 3: Figure S3, Additional file 4: Figure S4; Table 1). This variant was deemed to be deleterious because it is (1) predicted to impair splicing of the predominantly expressed transcript NM_000388 [32–34] and (2) absent from population genomic databases ExAC and gnomAD (Tables 1, Additional file 7: S3). This variant would be predicted to cause skipping of exon 6 and premature termination (p. Val537Metfs*49) prior to the receptor’s seven transmembrane spanning domain, similar to a previous example from the *CaSR* locus [35]. Of note, this variant would be predicted to cause a missense variant in the alternative transcript NM_001178065, which does encode a functional calcium sensing receptor but has only the third highest mRNA expression in the kidney relative to other alternative *CaSR* transcripts [32–34]. This missense variant was deemed deleterious in only one of three in silico prediction tools (Table 1). Sanger sequencing confirmed segregation of this variant in both affected siblings and lack of segregation with the unaffected father (Fig. 2). This variant, therefore, met the ACMG criteria [36] for a likely pathogenic variant.

**Table 1:** Genetic description and clinical data of family with dominant calcium-sensing receptor (*CaSR*) variant

Family ID/individuals	Age of onset (years)	NL Episodes	Stone composition (age at stone retrieval in years)	Variant genomic change^†^	Total allele frequency in ExAC, gnomAD v2.1.1, gnomAD v3.1.1 databases (H, het, total)	Canonical transcript NM_000388 Predicted Effect	Alternative transcript NM_001178065 predicted effect
KS72							
II 3	18	2	CaOxMo 56%, CaOxDi 41%, Struvite 3% (18)	GRCh37 Chr3:122000958A>G	Absent	c.1609-2A>G	c.1637A>G
				GRCh38 Chr3:12228211A>G		Splice Site Effect (MaxEnt -100%, NNSPLICE -100%, SSF -100%)	p.Gln546Arg (SIFT Tol 0.27; MT Del; PP2 Ben 0.032
II 4	19	2	CaOxMo 55%, CaOxDi 40%, Struvite 5% (19)				

*CaOxMo* Calcium oxalate monohydrate, *CaOxDi* Calcium oxalate dehydrate, *ID* identification code, *H* Homozygous subjects, *het* Heterozygous subjects, *MaxEnt* Maximum entropy splice site scoring software, *MT* “MutationTaster” prediction score, *NL* nephrolithiasis, *NNSSPLICE* Splice site prediction by neural network software, *PP2* PolyPhen-2 prediction score, *SIFT* “Sorting Tolerant From Intolerant” prediction score, *SSF* splice site finder software, *total* total alleles

^†^This variant was submitted from an independent case to ClinVar (RCV001059152.1) during the current study

In family KS-6, four family members had a history of urinary stones: II-2 (age of onset 18 years), the identical twins II-4 and II-5 (age of onset 17 years), and their mother I-2 (Additional file 2: Figure S2A, Additional file 6: Table S2). Stone composition analysis revealed predominantly calcium oxalate stones in all three affected siblings (Additional file 6: Table S2). All four affected family members report multiple NL recurrences since recruitment. Targeted sequencing detected a rare splice site variant (GRCh37 Chr3:122000929G >C GRCh38 Chr3:122282082G >C NM_000388:c.1609-31G >C NM_001178065:c.1609-1G>C) in *CaSR* (Additional file 6: Table S2; Additional file 2: Figure S2A, Additional file 3: S3). This variant is not predicted to effect splicing of the canonical transcript NM_000388, but rather impact an essential acceptor splice site position of exon 6 for the non-canonical transcript NM_001178065. This latter effect would result in premature termination (p.Pro537Metfs*49). This variant is rare in genome population databases overall. However, there is one homozygote and five heterozygotes present in the South Asian populations of these databases with no alleles identified in other populations (Additional file 6: Tables S2, Additional file 7: S3). Segregation analysis demonstrated that the variant was detected in all three affected siblings and not an unaffected sibling II-3 (Additional file 2: Figure S2A). The variant was not detected in the unaffected father, suggesting it may have been inherited from the affected mother. Because the biological importance of the alternative transcript NM_001178065 remains unclear, this variant was deemed to have unknown significance based on ACMG criteria [36].

In family KS-71, there were three affected family members (Additional file 2: Figure S2B; Additional file 6: Table S2). Subject II-5 had calcium oxalate NL beginning at age 20 years and had four NL episodes (Additional file 6: Table S2). Exome sequence data from this subject revealed the same rare splice site variant of unknown significance (NM_000388:c.1609-31G >C NM_001178065:c.1609-1G>C) in *CaSR* as was detected in family KS-6 (Additional file 6: Table S2). Additional DNA samples were not available from other family members for segregation analysis.

## Discussion

Nephrolithiasis affects 1 in 11 individuals worldwide. While NL has been associated with common *CaSR* variants [12–27], only specific rare inactivating CaSR variants in European and Australian kindreds cause NL [28–30]. These rare inactivating NL-associated variants are critical to discover and investigate, as they suggest the functions of the calcium sensing receptor in serum calcium homeostasis and parathyroid hormone regulation can be uncoupled from its role in renal calcium excretion. Discovery of novel NL-associated alleles can, therefore, provide a deeper understanding of the role of *CaSR* in calcium handling and kidney stone disease. For these reasons, the *CaSR* locus was sequenced in a cohort of 57 Pakistani families with pediatric NL, revealing rare loss-of-function variants in Pakistani kindreds with pediatric nephrolithiasis.

First, we detected a novel heterozygous and likely pathogenic splice variant (NM_000388:c.1609-2A>G) in *CaSR* in one family (KS-72) with pediatric calcium oxalate stones. This variant would be predicted to cause loss-of-function. NL has been observed in three atypical adult cases with dominant inactivating variants (K336del, L650P, F881L), hypercalcemia, and paradoxically familial hypercalciuria [29, 30]. These variants are spatially distinct and are predicted to impact the extracellular receptor domain, the transmembrane domain, or the intracellular C-terminal tail of the calcium sensing receptor, respectively. The variant in KS-72 is predicted to cause premature truncation of the receptor prior to its seven transmembrane spanning domain, which would be potentially more deleterious than the previous three variants identified in adult patients. A limitation of our study was that serum and urinary laboratory studies (e.g. PTH levels, serum calcium or phosphorous levels, urinary calcium excretion) to further characterize the NL-associated disease in KS-72 and other subjects were not available, because the primary physicians did not obtain these routinely during their clinical evaluation and because patients were not readily available to return to the clinical laboratory. Nevertheless, this variant was reported in ClinVar (VCV000854168.2) in a patient with a calcium disorder while our study was in progress. Overall, the novel variant expands the connection between rare inactivating *CaSR* variants and nephrolithiasis.

Secondly, we identified a splice variant of unknown significance in an alternative transcript (NM_001178065:c.1609-1G>C) in two additional families. Intriguingly, this variant would also be predicted to cause skipping of exon 6 in the alternative transcript and premature truncation prior to the receptor’s transmembrane domain. Alternatively, this variant may lead to further preference of the 5’ splice site of the canonical transcript NM_000388 exon 6. While aspects of our variant analysis would suggest this is a deleterious variant, further functional studies are warranted to understand if this alternative transcript plays an important or different role in calcium sensing relative to the canonical transcript (e.g. isoform-specific mouse knockouts).

Overall, our study reveals a novel rare *CaSR* variant associated with nephrolithiasis and suggests that specific inactivating variants in *CaSR* may have distinct effects on its role in calcium homeostasis.

## Supplementary Information

**Additional file 1:** Flow diagram of genetic variant filtering analysis.

**Additional file 2:** Pedigree structure of Pakistani NL families with CaSR VUS.

**Additional file 3:** Comparison of the canonical and alternative CaSR transcripts.

**Additional file 4:** Impact of CaSR variant in family KS-72 on the canonical and alternative CaSR transcripts.

**Additional file 5:** Primers sequence used for CaSR screening in nephrolithiasis patients.

**Additional file 6:** Genetic description and clinical data of families with calcium-sensing receptor (CaSR) variants of unknown significance.

**Additional file 7:** Allele frequency of NL-associated rare CaSR variants in South Asian and other populations of public genome databases.

## Data Availability

The datasets used and/or analysed during the current study are available from the corresponding author on reasonable request.

## Abbreviations

NL: Nephrolithiasis
*CaSR*: Calcium sensing receptor gene
