# Supplementary material for: Sequencing the CaSR locus in Pakistani stone formers reveals a novel loss-of-function variant atypically associated with nephrolithiasis
Source: BMC Med Genomics. 2021 Nov 12;14:266. doi: 10.1186/s12920-021-01116-5 (PMC8588693; doi:10.1186/s12920-021-01116-5)
Supplement: Supplementary file 1 — Additional file 1: Flow diagram of genetic variant filtering analysis. [file 12920_2021_1116_MOESM1_ESM.pdf]

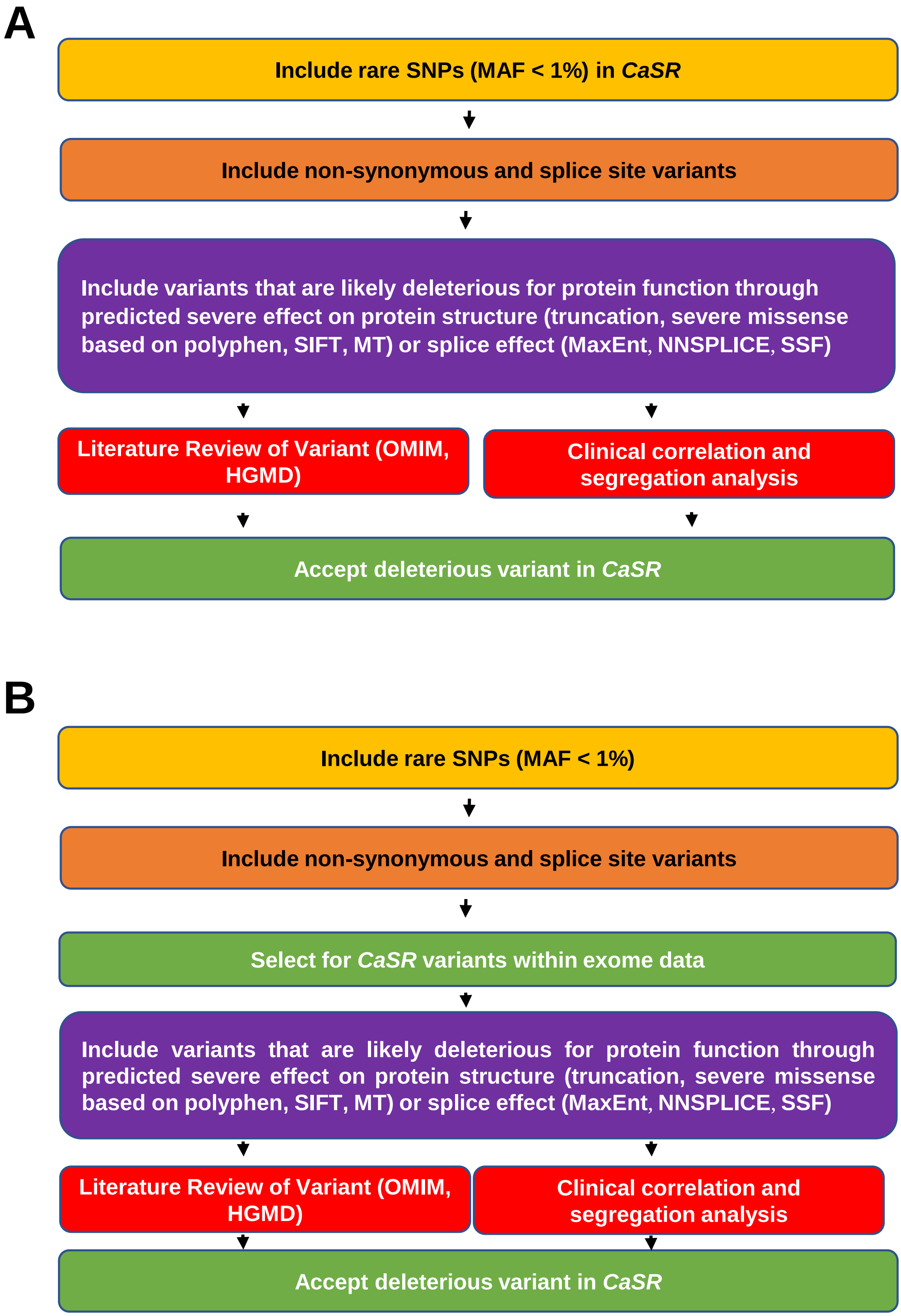

Supplementary Figure S1. Flow diagram of genetic variant filtering analysis. Variant filtering approach for screening of variants in *CaSR* gene are shown for Sanger sequencing data (A) and exome data analysis (B). Other established known NL disease gene loci were also interrogated in exome data as in (B) to exclude variants in other known disease genes in subjects with a *CASR* variant.
