# Supplementary material for: Sequencing the CaSR locus in Pakistani stone formers reveals a novel loss-of-function variant atypically associated with nephrolithiasis
Source: BMC Med Genomics. 2021 Nov 12;14:266. doi: 10.1186/s12920-021-01116-5 (PMC8588693; doi:10.1186/s12920-021-01116-5)
Supplement: Supplementary file 2 — Additional file 2: Pedigree structure of Pakistani NL families with CaSR VUS. [file 12920_2021_1116_MOESM2_ESM.pdf]

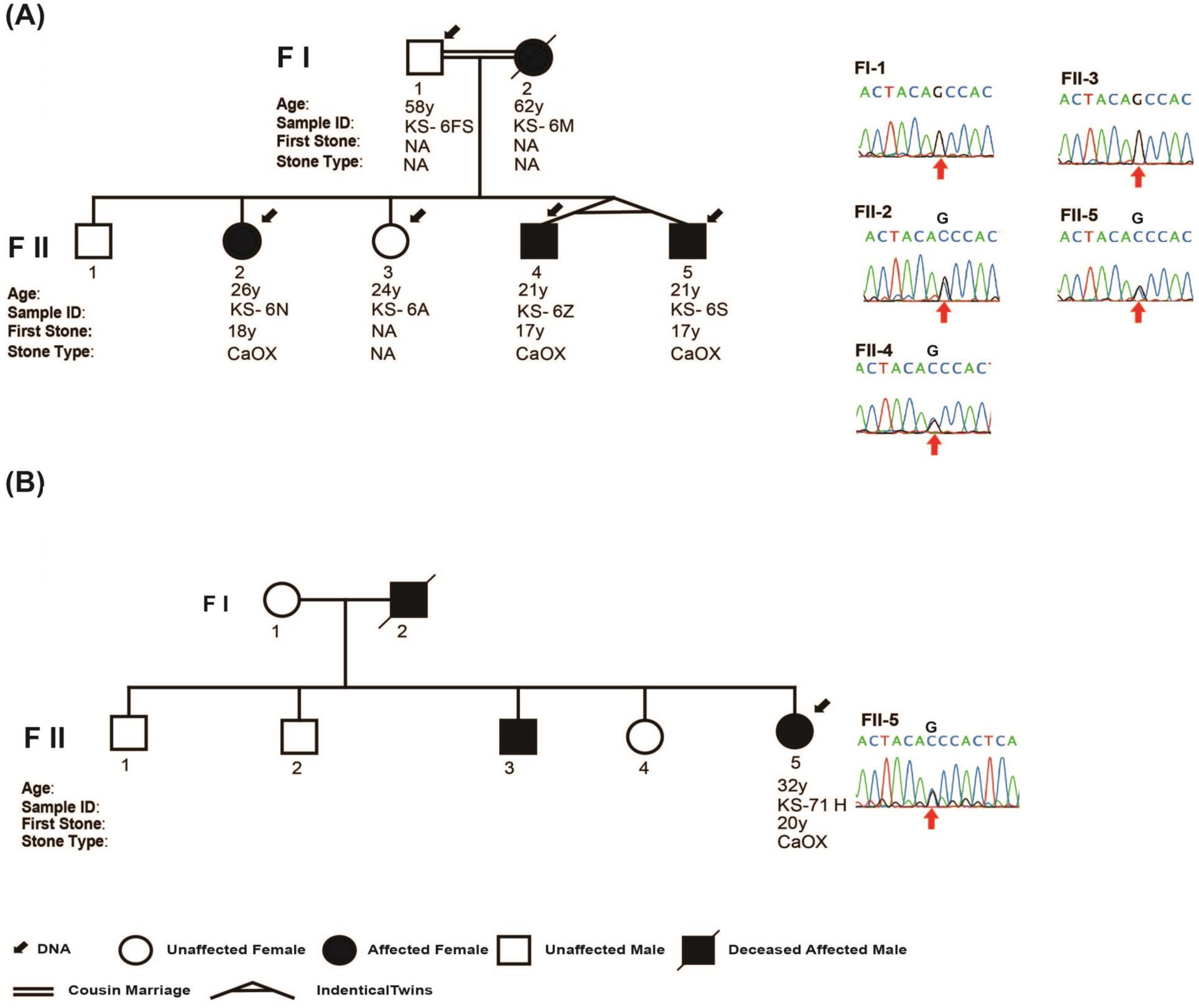

Supplementary Figure S2. Pedigree structure of Pakistani NL families with *CaSR* VUS. The pedigrees for families KS-6 (**A**) and KS-71 (**B**) are shown. Segregation of the *CaSR* variant of unknown significance (GRCh37 Chr3:122000929G>C; GRCh38 Chr3:122282082G>C; NM_000388:c.1609-31G>C; NM_001178065:c.1609-1G>C) of each family by Sanger sequencing chromatograms. Clinical data is shown below each symbol, and legend is provided at bottom for pedigrees.
