# Supplementary material for: Sequencing the CaSR locus in Pakistani stone formers reveals a novel loss-of-function variant atypically associated with nephrolithiasis
Source: BMC Med Genomics. 2021 Nov 12;14:266. doi: 10.1186/s12920-021-01116-5 (PMC8588693; doi:10.1186/s12920-021-01116-5)
Supplement: Supplementary file 3 — Additional file 3: Comparison of the canonical and alternative CaSR transcripts. [file 12920_2021_1116_MOESM3_ESM.pdf]

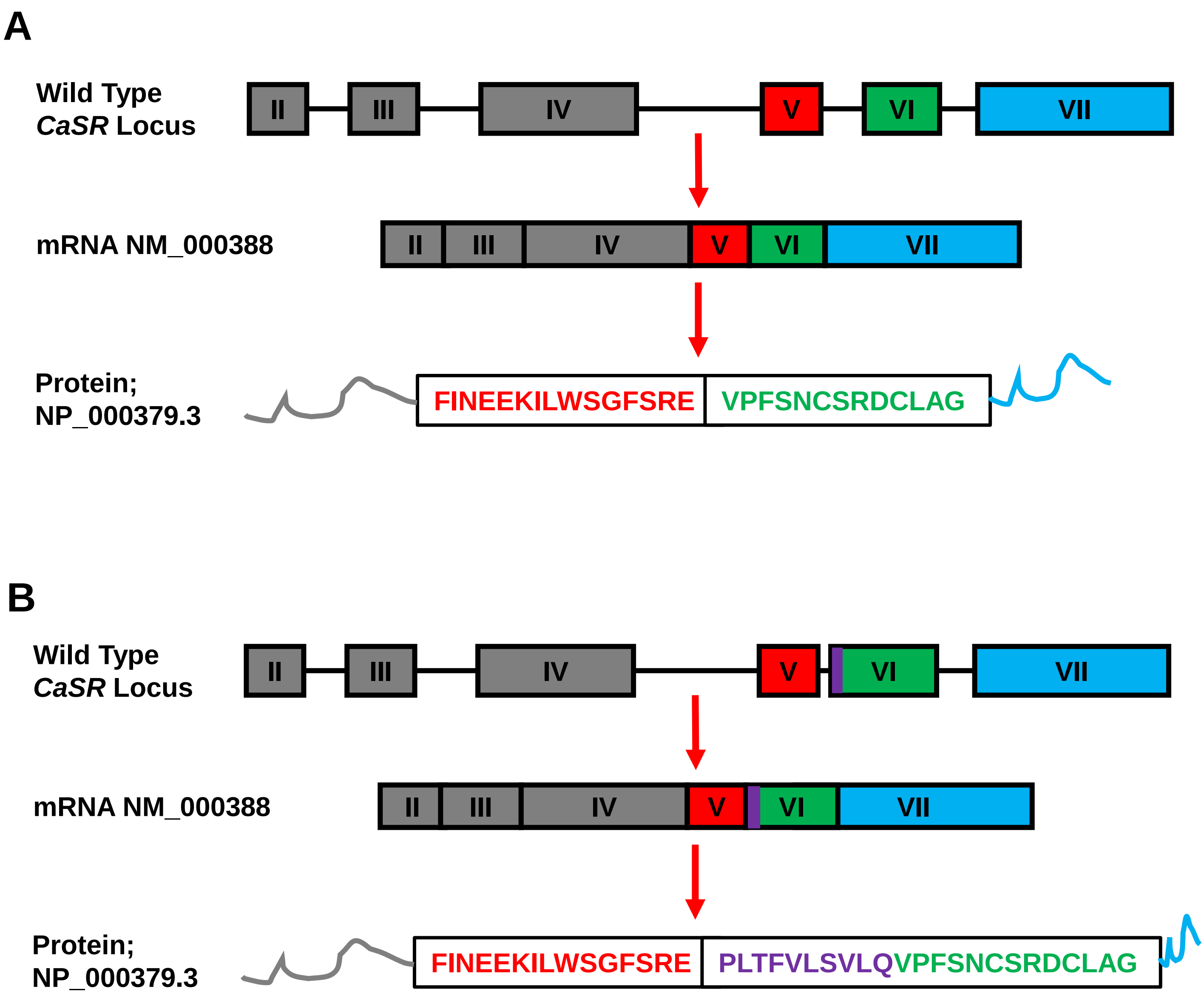

Supplementary Figure S3. Comparison of the canonical and alternative *CaSR* transcripts. *CaSR* transcripts NM_000388 and NM_001178065 are compared in (A) and (B), respectively. The loci encode splice variants of exon 6 with an additional 30 nucleotides incorporated into NM_001178065, which encodes an additional 10 amino acids (**purple**).
