# Supplementary material for: Sequencing the CaSR locus in Pakistani stone formers reveals a novel loss-of-function variant atypically associated with nephrolithiasis
Source: BMC Med Genomics. 2021 Nov 12;14:266. doi: 10.1186/s12920-021-01116-5 (PMC8588693; doi:10.1186/s12920-021-01116-5)
Supplement: Supplementary file 4 — Additional file 4: Impact of CaSR variant in family KS-72 on the canonical and alternative CaSR transcripts. [file 12920_2021_1116_MOESM4_ESM.pdf]

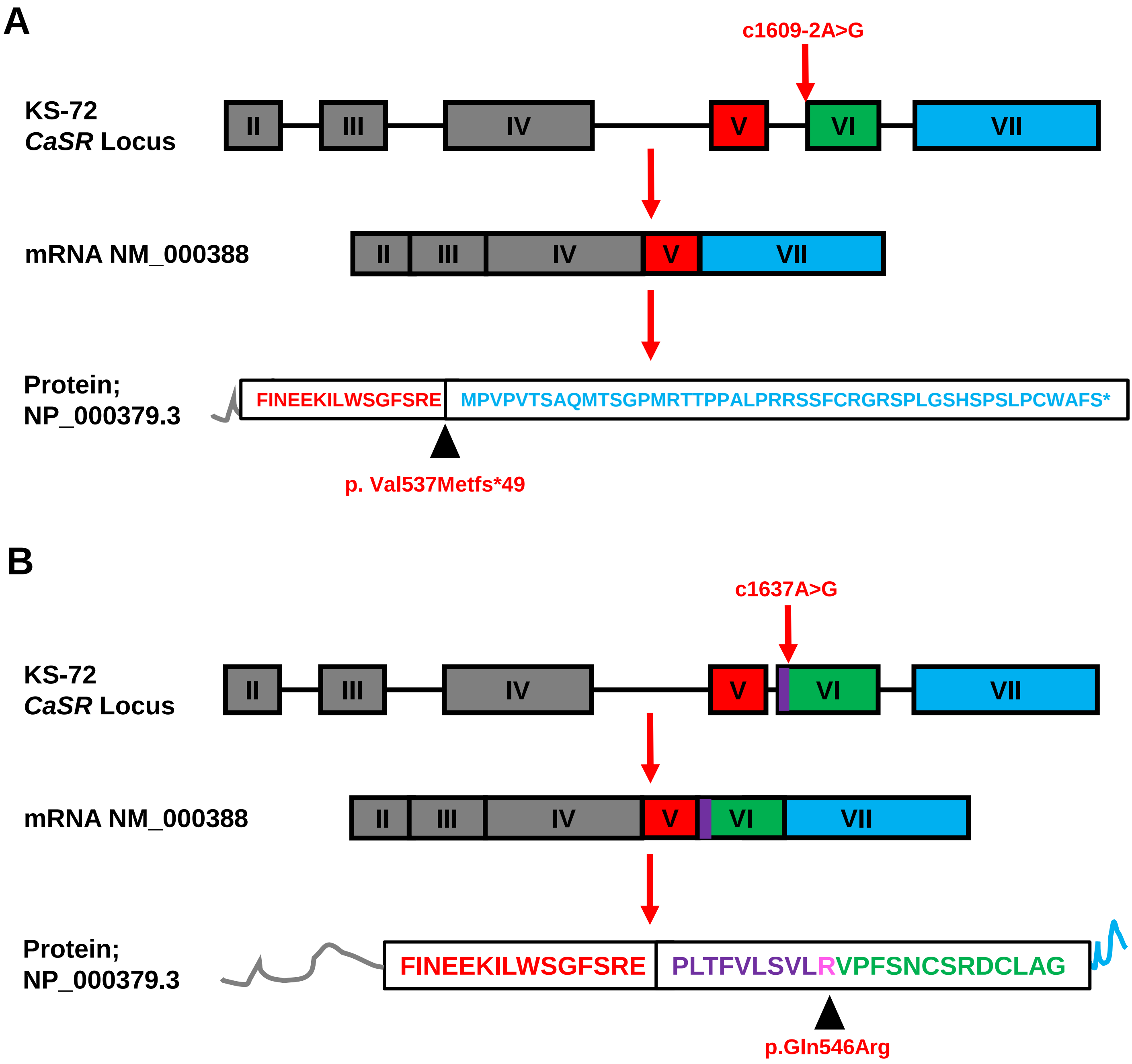

Supplementary Figure S4. Impact of *CaSR* variant in family KS-72 on the canonical and alternative *CaSR* transcripts.

The distinct impact of the *CaSR* variant in KS-72 is demonstrated in *CaSR* transcripts NM_000388 (A) and NM_001178065 (B). The variant impacts the acceptor splice site of exon 6 in the canonical transcript (A). In contrast, it is predicted to cause a missense variant in the coding region of the alternative transcript (B).
