# Supplementary material for: Sequencing the CaSR locus in Pakistani stone formers reveals a novel loss-of-function variant atypically associated with nephrolithiasis
Source: BMC Med Genomics. 2021 Nov 12;14:266. doi: 10.1186/s12920-021-01116-5 (PMC8588693; doi:10.1186/s12920-021-01116-5)
Supplement: Supplementary file 5 — Additional file 5: Primers sequence used for CaSR screening in nephrolithiasis patients. [file 12920_2021_1116_MOESM5_ESM.pdf]

Supplementary Table S1. Primers sequence used for *CaSR* screening in nephrolithiasis patients.

**Primer ID**	**Sequence**<br>**5' to 3'**	**Tm (C°)**
CASR1	GCCCACCCAAAGGAGTATG<br>AAGCTTAGGGCGCTTACGAC	60
CASR2	GCCACCTTAGTTGCAGTGGT<br>GTTTGGTGCAGCTTTTCTCC	59
CASR3a	CCAGCTTTGCCAGGTCTTTA<br>TTTGTGCCAGAGATGGGAAT	60
CASR3b	TGCTGGGGCTCTTCTACATT<br>TGGATTTGAGAGGTGGGATT	59
CASR4a	GGCTCACTCAGCACCTCTTC<br>TACTGAGGCATGGCGATCA	61
CASR4b	ATTGTCCGGCGCAATATC<br>GCAGCCCAACTCTGCTTTAT	59
CASR5	CAGGGCACAGCCTACCTAAT<br>AAGCCCAGCACAGTTTCCT	59
CASR6	GCCCAAACTCCTCCCTCTTA<br>CTTCCATGGGCTTCACTGAC	61
CASR7a	CCACCACCACATGTACACTCA<br>GCGGTGTAGAGCCAGATCAC	60
CASR7b	CTGCAGTTCCTGCTGGTTTT<br>CTTGCTGCTGATGGAGGAG	60
CASR7c	GCAGCTCACGCTTTCAAGG<br>ACCCCAAGAAACCTCTCTGC	62
CASR7d	TTCTCTGATGGCCAGTGATG<br>CCATGAAACTCTTGGGAGCTA	59

ID, identification code ; Tm, Primer melting temperature, 5' to 3' direction of primer synthesis
