# Supplementary material for: Sequencing the CaSR locus in Pakistani stone formers reveals a novel loss-of-function variant atypically associated with nephrolithiasis
Source: BMC Med Genomics. 2021 Nov 12;14:266. doi: 10.1186/s12920-021-01116-5 (PMC8588693; doi:10.1186/s12920-021-01116-5)
Supplement: Supplementary file 6 — Additional file 6: Genetic description and clinical data of families with calcium-sensing receptor (CaSR) variants of unknown significance. [file 12920_2021_1116_MOESM6_ESM.pdf]

Supplementary Table S2. Genetic description and clinical data of families with calcium-sensing receptor (*CaSR*) variants of unknown significance.

Family ID/Individuals	Age of Onset (years)	NL Episodes	Stone Composition (Age at stone retrieval in years)	Variant Genomic Change†	Total allele frequency in ExAC, gnomAD v2.1.1, gnomAD v3.1.1 databases (H, het, total)	Canonical Transcript NM_000388 Predicted Effect	Alternative Transcript NM_001178065 Predicted Effect
KS6							
II 2	18	3	CaOxMo 59%, CaOxDi 37%, Struvite 4% (20)	GRCh37 Chr3:122000929G>C<br>GRCh38 Chr3:122282082G>C	1/7/525072	c.1609-31G>C<br>No predicted splice effect	c.1609-1G>C<br>Splice Site Effect (MaxEnt -100%, NNSPLICE 0%, SSF -100%)
II 4	17	2	CaOxMo 58%, CaOxDi 36%, Struvite 6% (21)				
II 5	17	2	CaOxMo 60%, CaOxDi 38%, Struvite 2% (21)				
KS71							
II 5	20	4	CaOxMo 56%, CaOxDi 41%, Struvite 3% (32)	GRCh37 Chr3:122000929G>C<br>GRCh38 Chr3:122282082G>C	1/7/525072	c.1609-31G>C<br>No predicted splice effect	c.1609-1G>C<br>Splice Site Effect (MaxEnt -100%, NNSPLICE 0%, SSF -100%)

Abbreviations: CaOxMo, Calcium oxalate monohydrate; CaOxDi, Calcium oxalate dihydrate; ID, identification code; H, Homozygous subjects; het, Heterozygous subjects; MaxEnt, Maximum entropy splice site scoring software; NL, nephrolithiasis; NNSSPLICE, Splice site prediction by neural network software; SSF, splice site finder software; total, total alleles.
