# Supplementary material for: Sequencing the CaSR locus in Pakistani stone formers reveals a novel loss-of-function variant atypically associated with nephrolithiasis
Source: BMC Med Genomics. 2021 Nov 12;14:266. doi: 10.1186/s12920-021-01116-5 (PMC8588693; doi:10.1186/s12920-021-01116-5)
Supplement: Supplementary file 7 — Additional file 7: Allele frequency of NL-associated rare CaSR variants in South Asian and other populations of public genome databases. [file 12920_2021_1116_MOESM7_ESM.pdf]

Supplementary Table S3. Allele frequency of NL-associated rare *CaSR* variants in South Asian and other populations of public genome databases.

**Exome/Genome Aggregation Database**	**Homozygous/Heterozygous/Total Alleles, Allele Frequency**			
	Chr3:122000958A>G (GRCh37/hg19) Chr3:12228211A>G (GRCh38/hg38) NM_000388:c.1609-2A>G		Chr3:122000929G>C (GRCh37/hg19) Chr3:122282082G>C (GRCh38/hg38) NM_000388:c.1609-31G>C NM_001178065:c.1609-1G>C	
	South Asian	All Other Ethnic or Regional Groups	South Asian	All Other Ethnic or Regional Groups
ExAC (GRCh37/hg19)	0/0/16506, AF = 0	0/0/104870, AF = 0	0/1/16506, AF = 0.0001156	0/0/104870, AF = 0
gnomAD v2.1.1 (GRCh37/hg19)	0/0/30614, AF = 0	0/0/220852, AF = 0	1/5/30614, AF = 0.0001087	0/0/220852, AF = 0
gnomAD v3.1.1 (GRCh38/hg38)	0/0/4836, AF = 0	0/0/147394, AF = 0	0/1/4836, AF = 0.0002068	0/0/147394, AF = 0
Total	0/0/51956, AF = 0	0/0/473116, AF = 0	1/7/51956, AF = 0.00013	0/0/473116, AF = 0

Abbreviations: AF, Allele Frequency.
